# Identification of Atypical *El Tor* *V. cholerae* O1 Ogawa Hosting SXT Element in Senegal, Africa

**DOI:** 10.3389/fmicb.2017.00748

**Published:** 2017-05-15

**Authors:** Bissoume Sambe-Ba, Mamadou H. Diallo, Abdoulaye Seck, Abdoul A. Wane, Guillaume Constantin de Magny, Cheikh S.-B. Boye, Ahmad I. Sow, Amy Gassama-Sow

**Affiliations:** ^1^Unité de Bactériologie Expérimentale, Institut Pasteur DakarDakar, Sénégal; ^2^Laboratoire de Microbiologie Fondamentale et Appliquée, Faculté de Médecine et Pharmacie et d’Odontologie, Université Cheikh Anta DiopDakar, Sénégal; ^3^Laboratoire de Biologie Médicale, Institut Pasteur de DakarDakar, Sénégal; ^4^UMR IRD 224 – CNRS 5290 – Université de Montpellier – MIGEVEC, Centre IRD de MontpellierMontpellier, France; ^5^Laboratoire de Bactériologie, Centre Hospitalier Universitaire National de FannDakar, Sénégal; ^6^Département de Génie Chimique et Biologie Appliquée, Ecole Supérieure Polytechnique, Université Cheikh Anta DiopDakar, Sénégal

**Keywords:** *Vibrio cholerae*, O1 virulence, antibioresistance, SXT element, Senegal

## Abstract

*Vibrio cholerae* O1 is the causative agent of cholera with classical and El Tor, two well-established biotypes. In last 20 years, hybrid strains of classical and El Tor and variant El Tor which carry classical *ctx*B have emerged worldwide. In 2004–2005, Senegal experienced major cholera epidemic with a number of cases totalling more than 31719 with approximately 458 fatal outcomes (CFR, 1.44%). In this retrospective study, fifty isolates out of a total of 403 *V. cholerae* biotype El Tor serovar Ogawa isolates from all areas in Senegal during the 2004–2005 cholera outbreak were randomly selected. Isolates were characterized using phenotypic and genotypic methods. The analysis of antibiotic resistance patterns revealed the predominance of the S-Su-TCY-Tsu phenotype (90% of isolates). The molecular characterization of antibiotic resistance revealed the presence of the SXT element, a self-transmissible chromosomally integrating element in all isolates. Most of *V. cholerae* isolates had an intact virulence cassette (86%) (*ctx*, *zot*, *ace* genes). All isolates tested gave amplification with primers for classical CT, and 10/50 (20%) of isolates carried classical and El Tor *ctx*B. The study reveals the presence of atypical *V. cholerae* O1 El Tor during cholera outbreak in Senegal in 2004–2005.

## Introduction

Cholera is an epidemic diarrheal disease caused by toxigenic *Vibrio cholerae*, serogroup O1 or O139. There are two biotypes in the serogroup O1, classical and El Tor. The seventh pandemic of cholera were due to *V. cholerae* O1, biotype El Tor, began in Celebes (Islands) in 1961 and spread in West African countries in the early 1970s while the fifth and the sixth pandemics of cholera were caused by the classical biotype (Kaper et al., 1995). For over a decade, Africa has been the continent most affected by cholera in terms of the number of individuals infected and the frequency of outbreaks recorded^1^. In 2004–2005, cholera outbreaks occurred in eight countries in Africa with 125,082 cases and 2,230 deaths, CFR 1.78 (WHO, 2006). However, in some areas, the CFR exceeded 10%. In Senegal, the cholera outbreak has caused 31,719 cases with approximately 458 deaths (CFR, 1.44%) and the most affected regions were Diourbel and Dakar; the index case was a young Guinean living in a populous district in Dakar (Manga et al., 2008). Smaller scale epidemics have been reported from 2006 to 2008. However, no cases have been reported since July 2010, this is probably due to the monitoring implemented by the Senegalese Ministry for Health (Global Task Force on Cholera Control, 2006).

The pathogenicity of *V. cholerae* O1 and O139 isolates depends on a combination of factors including the coordinated expression of virulence factors, and the secretion of cholera toxin (CT). Molecular analysis of *V. cholerae* revealed the presence of two genetic elements in the genome of pathogenic strains: the lysogenic bacteriophage (CTXø), which hosts at least six genes including toxin genes: (*ctxAB*, *ace*, and *zot* encoding, respectively A and B subunits CT, accessory enterotoxin, and zonula occludens toxin), and the *Vibrio cholerae* pathogenicity island (VPI), which carries genes for the pilus colonization factor, toxin coregulated pilus (TCP) (Pearson et al., 1993; Waldor et al., 1997). Strains named “atypical El Tor” have traits of both classical and El Tor (Nair et al., 2002). Recently, several atypical El Tor strains have been reported, including Matlab variants (Safa et al., 2006), Mozambique variants (Ansaruzzaman et al., 2004), altered El Tor (Nair et al., 2006), and hybrid El Tor strains, harboring the classical CT allele *ctxB1* (Goel et al., 2008; Safa et al., 2008). Safa et al. (2010) proposed the term “atypical El Tor” for all *V. cholerae* O1 El Tor that harbor classical traits.

*Vibrio cholerae* O1 strains isolated in Africa are known to be resistant to many antibiotics (Ceccarelli et al., 2006; Quilici et al., 2010). Drug-resistance of bacteria is mainly linked to the mobilization and the dissemination of resistance genes through genetic determinants such as plasmids, integrons, and transposons. The SXT element is a self-transmissible mobile genetic element belonging to the family of integrating and conjugative elements (ICEs) that originally was discovered in a *V. cholerae* O139 isolate from India (MO10) which is resistant to streptomycin (Sm), trimethoprim (Tm), sulfamethoxazole (Su), and chloramphenicol (Wozniak et al., 2009; Ceccarelli et al., 2011b). The SXT element is always integrated into the 5′ end of the chromosomal gene *prfC* and able to replicate with the host chromosome (Waldor et al., 1997). Capture and spread of antibiotic resistance determinants by integrons is an effective route of antimicrobial resistance dissemination among Gram-negative bacteria (Mazel, 2006). Several of integrons have been described based on integrase gene. The class 1 integron is widely spread among *V. cholerae* isolates with various types of resistance gene cassettes (Dalsgaard et al., 2001; Ceccarelli et al., 2006). Class 4 integron named superintegrons is a component SXT element found in several bacteria in particular in *V. cholerae*. Superintegrons are the ancestors of multiresistant integrons (Mazel, 2006). Integrons and ICEs have been found in *V. cholerae* isolated in Mozambique, Iran, and India and they have largely contributed to the spread of antibiotic resistance (Amita et al., 2003; Adabi et al., 2009; Pugliese et al., 2009).

In Senegal, there is little data available on the genetic determinants of virulence and antibiotic-resistance among epidemic *V. cholerae* isolates. Despite the description of multiresistant isolates during the lastest cholera outbreaks in Senegal in 1994 (Aidara et al., 1998) and in 2004–2006 (Manga et al., 2008), the molecular mechanisms of antibiotic resistance have never been studied.

The objective of this work was to characterize the genetic determinants of virulence and antibiotic-resistance in *V. cholerae* O1 isolated during the latest cholera outbreak in Senegal.

## Materials and Methods

### Bacterial Isolates

**FIGURE 1:**
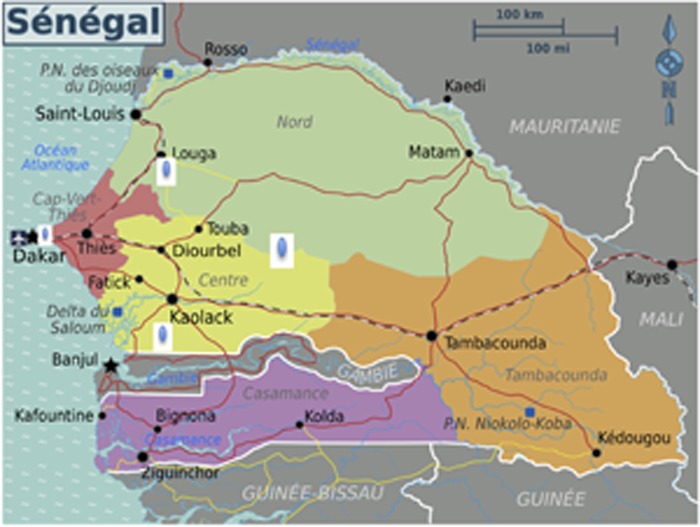
**Origin of isolates (Dakar: 39; Diourbel: 09; Kaolack: 01; Louga: 01)**.

A total of 403 *V. cholerae* serogroup O1 strains were isolated in different areas in Senegal between November 2004 to May 2005 from patients with acute diarrhea. A sampling of fifty isolates were randomly selected to represent the most affected population: Dakar (39); Diourbel (09); Kaolack (01); Louga (01) (**Figure 1**).

All isolates were identified with the API 20E (Biomérieux, Marcy l’Etoile, France) and serotyped using anti-Ogawa, anti-Inaba antisera.

### Susceptibility to Antibiotics

Antimicrobial susceptibility testing was performed using the Kirby Bauer disk diffusion method on Müller Hinton agar. The following antibiotics were tested: ampicillin (AM, 10 μg), amoxicillin-clavulanic acid (AMC, 20 μg/10 μg), cefotaxime (CTX, 30 μg), streptomycin (S, 10 μg), tetracycline (TCY, 30 UI), chloramphenicol (CHL, 30 μg), nalidixic acid (NA, 30 μg), pefloxacin (5 μg), trimethoprim (T, 5 μg), sulfamethoxazole (Su, 200 μg), trimethoprim-sulfamethoxazole (TSu, 1.25 μg/23.25 μg). The diameter of inhibition zones was interpreted following the CLSI recommendations for enterobacteria^2^.

### DNA Extraction

Total DNA was obtained by using QIAamp DNA Mini Kit (Qiagen SA, Courtaboeuf, France).

### PCR Amplifications

#### Detection of Antibiotic Resistance and Virulence Molecular Markers

PCR analysis was performed for detection of genetic determinants of antibiotic resistance and virulence. Amplification was carried out and virulence with primers described elsewhere (Ploy et al., 2000; Hochhut et al., 2001; Shi et al., 2006), and GenBank accession number AF 099172. For the detection of virulence genes, primers used as those described by Ogawa et al. (1990), Keasler (1993), and Shi et al. (1998) (**Table 1**) and GenBank accession numbers (AF262318, GQ485654). Chromosomal integration was detected by amplification of the right SXT element chromosome junction (*attP-prfC* gene sequence) (**Table 1**).

**Table 1:** Primers used for this study.

Primer sequence (5′–3′)	Primers	Target gene	Reference
ACA TGT GAT GGC GAC GCA CGA	intI1L	*intI1*	Ploy et al., 2000
ATT TCT GTC CTG GCT GGC GA	intI1R		
CAC GGA TAT GCG ACA AAA AGG T	intI2L	*intI2*	Ploy et al., 2000
GTA GCA AAC GAG TGA CGA AAT G	intI2R		
GCC TCC GGC AGC GAC TTT CAG	intI3L	*intI3*	Ploy et al., 2000
ACG GATCTGCCAAACCTGACT	intI3R		
GTG TTC GCG AAT TTA TGC	Int4-1	*intI4*	Shi et al., 2006
ACG GGA TAA TGG GCT TAA	Int4-2		
GCT GGA TAG GTT AAG GGC GG	SXT1	*int_SXT_*	Hochhut and Waldor, 1999
CTC TAT GGG CAC TGT CCA CAT TG	SXT2		
CAA GCG GAA AAA AAT CCA TA	SXT-R1F	*SXT prfC*	Pugliese et al., 2009
AGAGTCAACTGCGGTCAGAG	SXT-R1R		
CTCAGACGGGATTTGTTAGGCACG	ctxA-1	*ctxA*	Keasler, 1993
TCTATCTCTGTAGCCCCTATTACG	ctxA-2		
GGG CGA GAA AGG ACG C	Zot-1	*zot*	Shi et al., 1998
CCT TGT AGC GGT AGC TCG	Zot-2		
TAA GGA TGT GCT TAT GAT GGA CAC CC	Ace-1	ace	Shi et al., 1998
CGT GAT GAA TAA AGA TAC TCA TAG G	Ace-2		
CAC GAT AAG AAA ACC GGT CAAGAG	TcpA-F	*tcp* (Classical)	Ogawa et al., 1990
ACC AAA TGC AAC GCC GAA TGG AGC	TcpA-R		
GAA GAA GTT TGT AAA AGA AGA ACA C	TcpA-F	*tcp* (El Tor)	Ogawa et al., 1990
GAA AGG ACC TTC TTT CAC GTT G	TcpA-R		
ATC AG TGA TTC AAT CAT TC	RstC-F	*RST*(Classical)	AF 262318
ATT TAAGAG TTG AGA GAG AT	RstC-R		
AGA ATG TCT TAT CAG CAT AC	RstET-F	*RST*(El Tor)	
TAG CCA CCC AAA GAA AGG CA	RstET-R		
GATGGCAGCTTGCCGCAACCTC	SXT-X	*int_SXT_*	This study
GGAATTCGGCAGTCAAGGCAGAGGGC	SXT-X-M	*int_SXT_*	This study
CATCAGAAGTATAGAAATCTGACTG	SXT-X-2	*int_SXT_*	This study
TGTACGATCATTGAAATAAAAAGACC	SXT-X-3	*int_SXT_*	This study
GGAATTCGCGTTGCTGATCCGCAGCTTT	SXT-1-M	*int_SXT_*	This study
CGGGATCCGTTGTAGACCAACTTTTAACGTATAC	SXT-I-3-M	*int_SXT_*	This study
CCAGCTATTGAGCTGATTGAACTG	SXT-I	*int_SXT_*	This study

All amplified DNA fragments were resolved by conventional electrophoresis in 1% agarose gel, stained with ethidium bromide and visualized under UV light.

#### *ctxB* Typing by MAMA PCR

Mismatch Amplification Mutation Assay (MAMA) based PCR was performed to detect the presence of *ctxB* classical and or El Tor biotype *V. cholerae* O1 isolates, using specific primers described elsewhere (Morita et al., 2008).

### Cloning and Sequencing

The integrase SXT fragment (*int_SXT_*) was purified with the QIAquick kit (Qiagen SA, Courtaboeuf, France), and cloned with the pGEMT vector (Promega, Madison, WI, USA), transformed into XL1-Blue competent cells (Stratagene, Garden Grove, CA, USA).

The insert of the recombinant plasmid was sequenced with dye terminator on ABI Prism automatic sequencer as described by the manufacturers. The sequences were analyzed by nucleotide BLAST search at the National Center for Biotechnology Information (NCBI) website^3^. Primers were used for cloning and sequencing are listed on **Table 1**.

### Conjugation Experiments

Conjugation experiments were used to transfer resistance determinants from *V. cholerae* O1 isolates into nalidixic acid-resistant *E. coli* C1 strain. Mating experiments were carried out by mixing volumes of Luria Bertani (LB) broth in a ratio 2:1 overnight culture of donor and recipient strains. The cultures were transferred to LB agar plates containing trimethoprim (32 μg/ml), sulfamethoxazole (160 μg/ml), and nalidixic acid (50 μg/ml). To confirm the transfer of antibiotic resistance genes, transconjugants were tested for sensitivity to antibiotic and by PCR.

This study was carried out in accordance with the recommendations of Senegalese Ethical Committee, with informed consent from all subjects. The isolates used in this study were taken for the purposes of research, and the protocol was approved by the Senegalese Ethical Committee (N°0046/MSAS/DRPS/CNERS) ^4^.

## Results

### Antimicrobial Susceptibility

In this retrospective study, we show that isolates were resistant to at least four antibiotics including streptomycin (S), trimethoprim (T), sulfamethoxazole (Su), trimethoprim-sulfamethoxazole (Tsu). The analysis of antibiotic resistance patterns reveals three phenotypes: S-Su-T-TSu (90%), S-Su-T-TSu-C (8%), S-Su-T-TSu-AM (2%). All isolates were resistant to trimethoprim-sulfamethoxazole but susceptible to tetracyclines.

### Detection of Genetic Determinants of Antimicrobial Resistance: Detection of Integrons, Resistance Genes, and SXT Element

All isolates were negative for class 1, 2, and 3 integrons. A 900 bp PCR product of the *intI4* gene was obtained for all isolates.

The amplification of SXT integrase revealed an amplicon size of 3 kb in all isolates, different from the expected size, i.e., 592 bp. The SXT integrase from senegalese isolates was identical to a fragment of *V. cholerae* KN14, isolated in Kenya GenBank accession number (AB535680). The integration of the SXT element in the chromosome was highlighted by the amplification of the right SXT element-chromosome junction (*attP*-*prf*C) which producted a 785 bp PCR product.

To identify genes hosted by the ICE circulating in Senegal, PCR analysis revealed the presence of the following antibiotic resistances genes (*sulI*, *floR*, *strA*, and *dfrA1*), except *dfr18* gene.

### Detection of Virulence Markers

**FIGURE 2:**
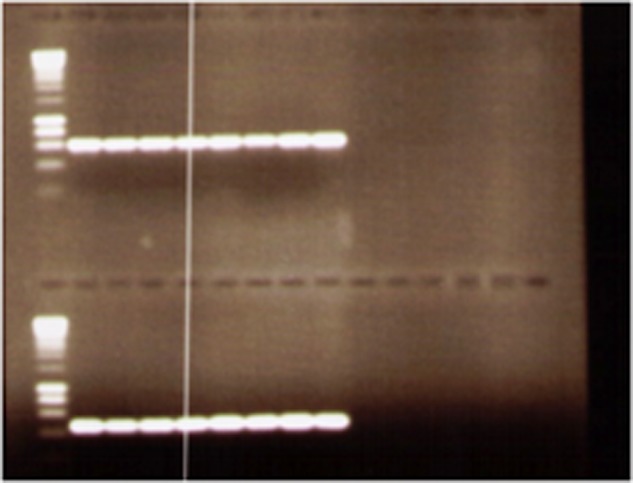
**Agarose gel electrophoresis of examples of PCR products of *Vibrio cholerae* O1 isolates using *tcpA* (classical and El Tor)**.

The *ctxB*, *zot, ace* genes in the CTX element were present, respectively, in 98, 92, and 88% of isolates. All isolates gave positive results for *tcpA* (classical and El Tor) (**Figure 2**) and for bacteriophage *rst*R repressor gene of the El Tor and classical types. The detection of virulence genes revealed the presence of the genome of filamentous bacteriophage CTXø.

### *ctxB* Typing

All isolates tested gave amplification with primers for classical CT allele *ctxB1*, and 10/50 (20%) of isolates gave amplicons with primers specific for classical and El Tor CT allele *ctxB1.*

### Conjugation Experiments

Mating experiments revealed transfer of the resistance determinants to chloramphenicol, streptomycin, sulfamethoxazole, and trimethoprim by conjugation. To identify a possible ICE-mediated resistance, transconjugants were tested for SXT-related elements. Antimicrobial susceptibility testing of *V. cholerae* transconjugants showed that resistance profiles were expressed by each of the transconjugants. The PCRs for detection of the *int_SXT_* element-integrase gene gave an amplicon of the same size of 3 Kbp.

## Discussion

Senegalese isolates are still susceptible to quinolones while resistance to nalidixic acid or reduced sensitivity to a fluoroquinolone was described in Africa and India (Quilici et al., 2010; Ismail et al., 2013; Kutar et al., 2013).

Our results showed that class 1, 2, and 3 integrons were not involved in the spread of resistance among Senegalese *V. cholerae* O1 isolates, even though they have been detected in Mozambican *V. cholerae* O1 isolates and other Gram-negative enteric bacteria in Senegal (Hochhut and Waldor, 1999; Dalsgaard et al., 2001; Gassama et al., 2004; Gassama-Sow et al., 2006). According to Mazel (2006), the *IntI4* gene associated with superintegron is characterized by a large number of gene cassettes, closely associated with genome evolution rather than the capture of antibiotic resistance.

All genes (*sulI*, *floR*, *strA*, and *dfrA1*) excepted *dfrA18 gene* were found in our isolates suggesting that the SXT isolated in Senegal is closely related to the SXT^ET^. Kenyan isolates also lacked the *dfrA18* gene (Kiiru et al., 2009). Since the emergence of SXT in *V. cholerae* O139, several studies on *V. cholerae* O1 have found this ICE as responsible for the dissemination of antibiotic resistance in Africa and Asia (Dalsgaard et al., 2001; Amita et al., 2003; Opintan et al., 2008; Adabi et al., 2009; Pugliese et al., 2009). ICEs of the SXT/R391 family are usually found in atypical O1 El Tor *V. cholerae* epidemic strains; they confer a narrow antibiotic resistance profile (Wozniak et al., 2009). Conjugation experiments revealed that although the isolates carry the SXT/R391-like elements which confers resistance to streptomycin, trimethoprim sulfamethoxazole and chloramphenicol, they lack multiple resistant integrons.

Further studies are needed to characterize and completely sequenced the SXT-related ICE in senegalese isolates.

*Vibrio cholerae* O1 strains isolated during the lastest outbreak (2004–2005) in Senegal were “atypical” as appointed by Safa et al. The presence of these atypical isolates may explain the disease severity After 2001, atypical *V. cholerae* O1 strains have emerged in India and spread worldwide, particularly in Africa. Indeed, atypical *V. cholerae* O1 strains were described in Mozambique (B33) (Ansaruzzaman et al., 2004), and Angola (Ceccarelli et al., 2011a). The appereance of atypical strains in Senegal is enigmatic, and suggest that probably these new strains followed the same West African path used by cholera to enter Africa in the early 1970s. The presence of int_sxt_ identical to a fragment of *V. cholerae* KN14 could confirm this hypothesis. The global replacement of El Tor prototype by atypical strains indicates the evolution of *V. cholerae* O1. Our study revealed that atypical strains are also in the process of replacing El Tor strains; this phenonenon has been described in Eastern Africa (Ceccarelli et al., 2011a). This global replacement is believed to be due to unknown environmental factors and phages contribution (Faruque and Mekalanos, 2012).

## Conclusion

Based to our results, atypical *V. cholerae* O1 El Tor strains were responsible for cholera outbreak in Senegal in 2004–2005.

According to our study integrons were not involved in the spread of resistance among senegalese isolates of *V. cholerae* O1 even though they have been detected in other Gram-negative enteric bacteria in Senegal. However, the detection of the SXT element in all isolates, irrespective of their resistance phenotypes, could have a clinical significance and should be monitored to avoid dissemination in other bacteria. The understanding of the basis of antimicrobial resistance patterns could inform guidelines for empirical treatment to reduce injudicious antimicrobial use. Further studies should be conducted to characterize the SXT element identified in Senegalese *V. cholerae* isolates. The genetic changes occurred in *V. cholerae* O1 El Tor strains need to be monitored to prevent severe cholera outbreaks in Africa.

## Author Contributions

BS-B participated in the molecular genetic studies and drafted the manuscript. MD carried out the molecular genetic studies. AW participated in the molecular genetic studies. AS participated on the identification of isolates. GC helped in drafting the manuscript. AIS helped on the collection and the identification of isolates in all sites. CB participated in the design and coordination of the study. AG-S designed the study, and wrote the manuscript. All authors read and approved the final manuscript.

## Conflict of Interest Statement

The authors declare that the research was conducted in the absence of any commercial or financial relationships that could be construed as a potential conflict of interest.

## Footnotes

1 http://www.who.int/wer

2 http://www.clsi.org/

3 https://blast.ncbi.nlm.nih.gov/Blast.cgi

4 www.healthresearchweb.org/fr/senegal/ethics_1057
